# Environmental suitability of Anopheles mosquito species in the Maekel region, Eritrea: species distribution modeling using maximum entropy

**DOI:** 10.1186/s12936-026-06042-4

**Published:** 2026-07-08

**Authors:** Filmon Gebreyesus Mebrahtu, Kibreab Tesfamichael Haile, Adam Mengesteab Teweldebirhan, Meron Berhe Tsegai, Selam Mihreteab, Araia Berhane

**Affiliations:** 1 Environmental Health Unit, Ministry of Health, Zoba Maekel Branch, Asmara, Eritrea; 2 Malaria Control Unit, Ministry of Health, Zoba Maekel Branch, Asmara, Eritrea; 3 Planning Unit, Ministry of Health, Zoba Maekel Branch, Asmara, Eritrea; 4 National Malaria Control Program, Communicable Diseases Control Division, Ministry of Health, Asmara, Eritrea; 5 National Communicable Disease Control Division, Ministry of Health, Asmara, Eritrea

## Abstract

**Introduction:**

Malaria, a significant global health challenge, is transmitted exclusively by Anopheles mosquitoes. The dependence of mosquito habitats on environmental conditions, coupled with the established link between malaria prevalence and habitat niches, highlights the dynamic interplay between mosquito ecology and environmental factors in malaria transmission. This study aims to determine the current and future environmental suitability of Anopheles mosquitoes in the Maekel region using species distribution modeling (SDM).

**Methodology:**

Species occurrence data, along with environmental and bioclimatic variables, were used to model habitat suitability using Maxent. Highly correlated variables, defined as those with a Pearson correlation coefficient exceeding 0.7, were excluded from the model. Future climate projections from the Coupled Model Intercomparison Project (CMIP-6) were used to project species distributions under two shared socioeconomic pathway scenarios: SSP370 and SSP585. Key environmental variables influencing species survival were identified through the jackknife test of variable importance. Model performance was evaluated via area under the curve (AUC) statistics.

**Results:**

A total of 94 larval breeding sites positive for *Anopheles* were identified across 24 villages. The most prevalent species, *Anopheles cinereus*, was identified in 20 villages, followed by *Anopheles demeilloni* and *Anopheles squamosus*. *Anopheles gambiae*, the primary malaria vector in Eritrea and Africa, was identified at 5 sites in 4 villages. Maxent modeling indicated that large areas of the Maekel region are highly suitable for Anopheles species, with projected increases in suitability by 2030 and 2050 under both climate change scenarios.

**Conclusion:**

Anopheles mosquitoes are well established across significant portions of the Maekel region, and their distribution is projected to expand in the future. Comprehensive nationwide studies are necessary to map species distributions and bionomics, enabling the refinement of malaria control strategies in response to the rapid expansion of Anopheles mosquitoes driven by climate change.

## Introduction

Malaria remains a major global health burden and is transmitted by the bite of female Anopheles mosquitoes. In 2023, malaria resulted in an estimated 263 million cases and 597,000 deaths, with the majority occurring in sub-Saharan Africa and among children under five years of age [1]. Efforts to control and eradicate malaria have focused primarily on the control of Anopheles mosquito vectors. Control measures, including larviciding, insecticide residual spraying, and the use of insecticide-treated bed nets, have been effective in reducing malaria transmission. Globally, 2.2 billion cases and 12.7 million deaths were averted between 2000 and 2023[1].

Emerging trends, such as rapid vector mutation, evolution of Anopheles mosquito bionomics, and climate change, threaten to reverse progress in malaria control. An invasive vector, *Anopheles stephensi*, originating from Southeast Asia, has been spreading across Africa since its landfall in Djibouti in 2012. This has exposed 126 million people in urban areas to malaria risk [2]. Climate change is also facilitating the spread of malaria into previously unaffected areas, as evidenced by recent outbreaks [1, 3, 4]. Mosquitoes are defeating aegis of altitude, ascending approximately 6.5 m annually [4–7].This has significant implications, as many urban settlements were historically established at relatively high altitudes to avoid mosquitoes. Consequently, millions of individuals with limited immunity to malaria in these areas may become vulnerable to malaria infection.

Species distribution models (SDMs) and disease risk maps have become vital tools for predicting such scenarios. SDMs visualize areas suitable for mosquito reproduction and malaria transmission. Highly accurate SDMs can assist malaria control programs in implementing targeted interventions, thereby enhancing preparedness for future malaria trends[8, 9]. Among the available SDM techniques, maximum entropy (Maxent) has gained prominence because of its simplicity, robustness, and accuracy [10]. Maxent can produce reliable projections using only species presence data, even with limited sample sizes.

In Eritrea, malaria transmission exhibits seasonal patterns, proceeding after the rainy season. Prevention efforts have achieved substantial reductions in malaria cases and deaths, with an 80% decrease since the 1998 peak outbreak [11]. However, Eritrea's vulnerability to climate change poses a risk to these gains. Currently, there are limited data on the distribution of Anopheles mosquitoes, the factors influencing their distribution, and future projections of their spread in Eritrea. Analyzing areas of potential habitat suitability for Anopheles mosquitoes is crucial for guiding malaria prevention and control efforts. Therefore, this study aims to assess the environmental suitability, influential climatic and environmental variables, and future projected distribution of Anopheles mosquitoes using Shared Socioeconomic Pathway (SSP) scenarios.

## Methods

### Study area

The study was conducted in the Maekel region, one of the six administrative zones of Eritrea. The region is divided into 16 administrative sub-zones and 83 villages. The Maekel region is situated in the Eritrean highlands, with an average altitude of 2219 m (range: 1307–2601 m) above sea level. The region have 1014 km^2^ surface area and is characterized by a humid‒arid subtropical climate with localized microclimates. The Maekel Region is the most densely populated region in Eritrea, with an average population density of 459 people per square kilometer (range: 46–5912) (Fig. 1)[12]. Malaria is largely absent in the region, although a few indigenous cases are reported annually.

**Fig. 1:**
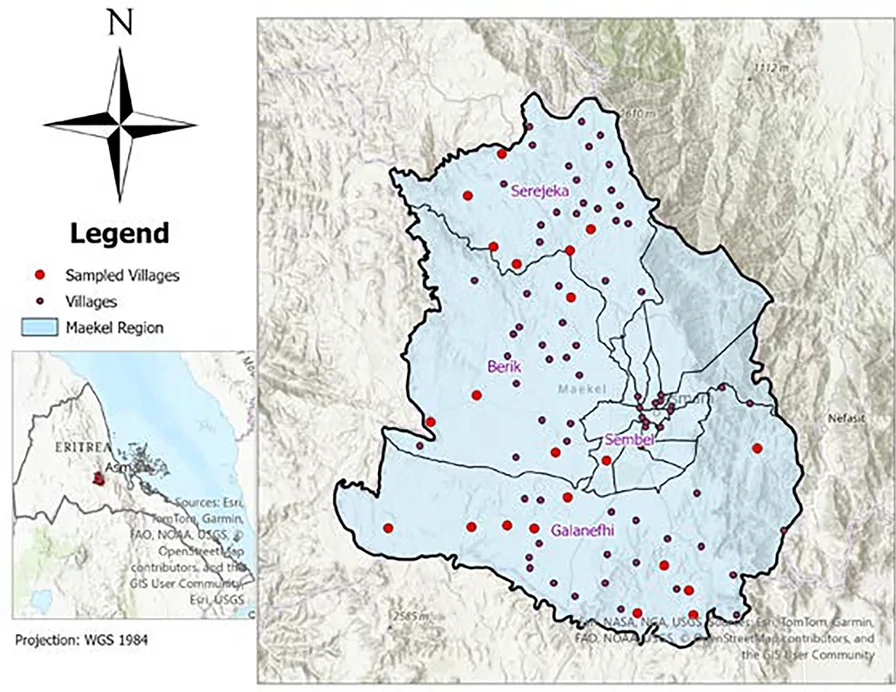
Maekel region

### Mosquito collection and identification

Larval collection was conducted by entomological experts at potential breeding sites from September 10^th^ to 30^th^, 2023. The collection time aligns with the end of the main rain season in the region. The study was conducted in 4 sub-zones out of 16 subzones in the Maekel region(Sembel, Serjaka, Berik, Gala-Nefhi). Anopheles species larvae were collected from breeding habitats using a WHO standard 350 ml dipper. Larvae were transported to the Mendefera Entomological Laboratory for rearing and identification. Species identification was performed morphologically using the standard taxonomic keys for the *Anopheles* of the Afrotropical Region by Gillies and Coetzee (1987) [13]. Specimens were examined under a dissecting microscope to identify diagnostic features such as the arrangement of setae and pigmentation patterns. The geographic positioning system (GPS) coordinates of each breeding site were recorded via Android phones with an open data kit (ODK) form. All the GPS coordinates were collected in the WGS1983 projection, with a minimum accuracy of 4 m.

Current bioclimatic data (1970–2010) were downloaded at a 30 arc-second (1 km^2^) resolution from WorldClim (V2.1) [14, 15]. Future bioclimatic data for two projection periods (2021–2040 and 2041–2060) and two shared socioeconomic pathway scenarios (SSP3-3.7 and SSP5-8.5) from the ACCESS-CM2 projection group[14] were obtained [16]. Elevation data were also downloaded from the same source [14]. Four additional variables, namely, aspect, slope, hill shade, and topographic wetness index, were derived from the elevation data. Pearson correlation tests were performed via the SDM toolbox v2.0 [17] in ArcMap v10.8 to assess correlations between variables. Variables with a correlation coefficient exceeding 0.7 (r > 0.7) were excluded from the modeling. Of the 24 initial variables (19 bioclimatic variables and 5 topographic variables), 14 were retained for the final model (Table 1).

**Table 1:** Variables included in modeling

Abbreviation	Descriptions
Bio1	Annual mean Temperature
Bio2	Mean diurnal range (mean of monthly temperature max–min)
Bio3	Isothermality (bio2/bio7) *100
Bio7	Temperature annual range (Bio5-Bio6)
Bio8	Mean temperature of wettest Quarter
Bio12	Annual precipitation
Bio13	precipitation of the wettest month
Bio14	precipitation of driest month
Bio18	precipitation of warmest quarter
Bio19	precipitation of coldest quarter
Aspect	Direction of the maximum gradient and relates to the degree of solar exposure. The aspect determines the effect of solar heating, air temperature, and moisture. Aspect ranges from 0^°^ to 360^°^ (0^°^ &360^°^ north, 90^°^ east, 180^°^ south and 270^°^ west)
Slope	The angle of ascent and descent for each pixel on a hillside. The lower the slope value the flatter the terrain; the higher the slope value, the stepper the terrain. Slope strongly influences overland and subsurface flow velocity, drainage, and water accumulation
Hill shade	Local areas with shadows thrown upon raised landscapes. The higher the shading values, the higher and warmer the surface (more exposure to sun rays); and the lower the hill shading values the darker and cooler the surface
Topographic wetness index	TWI is an estimate of the predicted water accumulation and is calculated as the natural logarithm of the ratio between the total catchment area and slope. High TWI values indicate converging terrains and valleys, while low values are typical steep and diverging terrains. TWI is a proxy for the depth of groundwater, soil pH, vegetation species richness, and soil organic matter

### Data modeling

All climatic data were clipped to the Maekel region boundaries and converted to ASCII raster format with the same grain size and WGS1983 projection. To enhance model performance, 10,000 randomly distributed background points were generated. To remove overrepresentation of the ecological area, rarefying of the presence data was performed at a 5 km distance. All Maxent feature types (linear, quadratic, product, threshold, hinge, and categorical) were evaluated during modeling. Regularization parameters of 1.5, 2, and 2.5 were used to minimize model overfitting. Model training and validation were performed via a cross-validation technique with 10 replicates. Jackknife tests and response curves were used to assess variable importance.

Model performance was evaluated via the area under the curve (AUC) of the receiver operating characteristic (ROC). An AUC value of 1 indicates perfect discrimination. The final species distribution maps were categorized into five suitability classes: very high (> 0.6), high (0.4–0.6), moderate (0.2–0.4), low (0.1–0.2), and very low (< 0.1) [18]. Data preparation was conducted via ESRI ArcMap version 10.8 [19], and modeling was performed using Maxent software version 3.4.4 [10].

## Results

A total of 94 larval breeding sites positive for *Anopheles* were identified across 24 villages. From these sites, immature stages were collected and reared to adulthood for morphological identification, resulting in the confirmation of seven distinct *Anopheles* species. *Anopheles cinereus* was the most common species, found in 20 villages, followed by *Anopheles demeilloni* (16 villages) and *Anopheles squamosus* (12 villages). *Anopheles gambiae*, the primary vector in Eritrea, was identified at 5 sites (Table 2).

**Table 2:** Anopheles species Identified

*Anopheles* species	Number of villages positive for anopheles	Positive breeding sites
*Anopheles cinereus*	20	28
*Anopheles demeilloni*	16	20
*Anopheles squamosus*	12	20
*Anopheles dthali*	7	11
*Anopheles gambiae*	4	5
*Anopheles pretoriensis*	2	3
*Anopheles salbi*	1	2

### Species distribution model

Species distribution models were constructed for *Anopheles cinereus*, *Anopheles demeilloni*, *Anopheles squamosus*, *Anopheles dthali*, and *Anopheles gambiae*. Models for *Anopheles pretorensis* and *Anopheles salbi* were not developed due to insufficient sample sizes after rarefying the presence points.

*Anopheles gambiae* were identified at five sites. Under current climatic conditions, large portions of the study area exhibit moderate and high suitability for *Anopheles gambiae*, with small areas in the southeast showing very high suitability. Under the SSP3-3.7 projection, areas of moderate suitability transition to high and very high suitability by 2030, particularly in the northern parts. This trend is further pronounced by 2050. Similar patterns of increased suitability are observed under the SSP5-8.5 scenario. The model demonstrated good performance, with training and test AUC values of 0.76. Jackknife tests revealed that hill shade and the topographic wetness index were the most influential variables for Anopheles gambiae habitat suitability.

*Anopheles cinereus* was the most widespread species and was identified at 28 sites. Under current climatic conditions, areas in the north, west, center, and southeast regions exhibit very high suitability, whereas the eastern region has very low potential. Under SSP3-3.7, areas of high suitability for Anopheles cinereus increase and expand westward by 2030, with further expansion and consolidation observed by 2050. A similar, but more pronounced, pattern of expansion is observed under SSP5-8.5. The model demonstrated good performance, with training and test AUC values of 0.843 and 0.695, respectively. The key influential variables included the topographic wetness index, precipitation in the driest month (Bio14), aspect, hill shade, mean diurnal temperature range (Bio2), and precipitation in the warmest month (Bio18).

*Anopheles demeilloni* was the second most common Anopheles mosquito and was identified at 20 sites across 16 villages. Large areas of the region exhibit very high and high suitability for *Anopheles demeilloni*. Under the SSP3-3.7 scenario, very high suitability areas are limited primarily to the east and center of the region by 2030. By 2050, very high suitability areas in the east revert to high suitability areas, and high suitability areas are concentrated in the southeast. Under SSP5-8.5, very high suitability areas are projected in the east, southeast, and southern fringes by 2030. By 2050, these areas show slight expansion. The model demonstrated good predictive performance, with an AUC of 0.77 (Training & Test). The key influential variables included hill shade, aspect, mean diurnal temperature range (Bio2), precipitation in the driest month (Bio14), topographic wetness index, and precipitation in the warmest month (Bio18).

*Anopheles squamosus* was identified at 20 sites across 12 villages. Current climate projections show very high suitability in the northwest and in patches in the central and south of the region. Under SSP3-3.7, high suitability areas transition to moderate suitability areas by 2030, while very high suitability areas in the center shrink, and those in the south parts will expand slightly (Table 3). The model demonstrated good to excellent predictive performance, with training and test AUC values of 0.80 and 0.95, respectively. The key influential variables included the topographic wetness index, precipitation in the driest month (Bio14), aspect, hill shade, slope, mean diurnal temperature range (Bio2), and precipitation in the warmest month (Bio18).

**Table 3:**
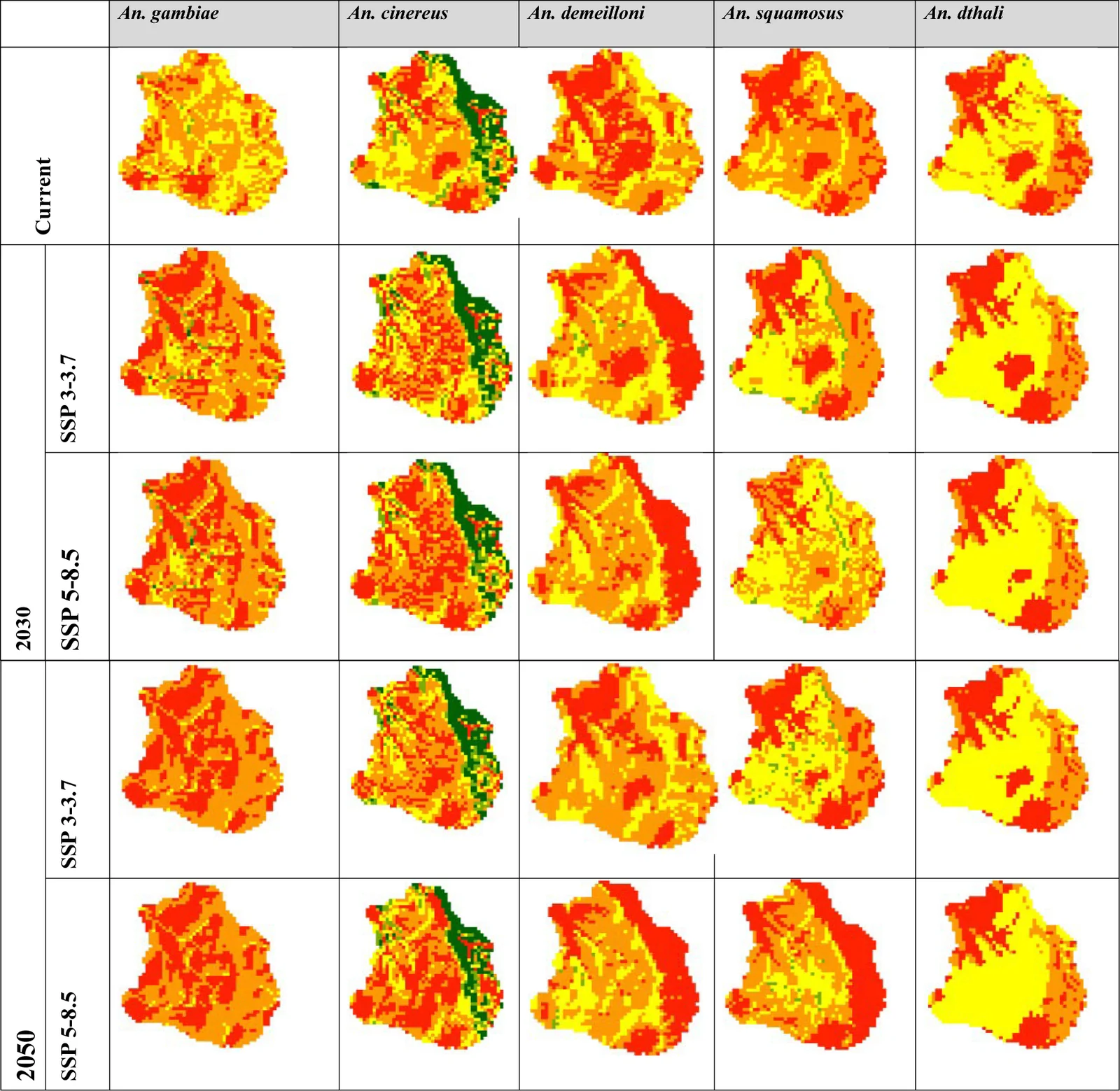
Current and future projections of Anopheles mosquitoes in Zoba Maekel

*Anopheles dthali* were identified at 11 sites across 7 villages. Currently, areas in the central, southeastern, and northwestern parts of the regions are highly suitable. A large diagonal region between the southeast and northwest regions shows moderate suitability. Similar patterns are projected under future climate conditions. The high-suitability area in the center shrinks by 2050 under SSP3-3.7 and disappears under SSP5-8.5. The other high-suitability areas remain stable under both scenarios and projection periods. The model demonstrated excellent performance, with training and test AUC values of 0.88 and 0.95, respectively. The key influential variables included the topographic wetness index, precipitation in the driest month (Bio14), aspect, hill shade, slope, mean diurnal temperature range (Bio2), and precipitation in the warmest month (Bio18).

## Discussion

This study aimed to analyze the potentially suitable areas and future expansion of Anopheles mosquitoes in the Maekel Region of Eritrea via Maxent. The results indicate that large areas of the Maekel region are suitable for Anopheles mosquitoes, specifically *Anopheles gambiae, Anopheles cinereus, Anopheles squamosus,* and *Anopheles demeilloni*. Further increases in suitability are projected under both the SSP3-3.7 and SSP5-8.5 scenarios.

*Anopheles gambiae* is associated with small areas of very high suitability, which are projected to expand in the future under both scenarios. Anopheles gambiae is known for its high adaptability to topographic and ecological factors, making it the most efficient malaria vector in Africa [20, 21]. In Eritrea, Anopheles gambiae is the primary malaria vector and is predominantly found in the malarious areas of the Gash Barka and Debub Regions. A previous study conducted in 2001–2002 found a vector density of 0.4% for *Anopheles gambiae* in Maekel Region [22]. The current study projects an increment to the areas of potential environmental suitability for *Anopheles gambiae*. Similar results have been reported by Olabimi et al., who concluded that *Anopheles gambiae* will continue to have large areas of high suitability [23]. The limited number of Anopheles gambiae in the current study supports the hypothesis that An.gambiae is starting to establish itself in the Maekel Region, driven by climate change facilitating range expansion.

The study area features large areas of very high and high suitability for *Anopheles cinereus* and *Anopheles dthali*. This high prevalence may be attributed to its affinity of *An. cinereus* for cooler, high-altitude environments compared to *An. gambiae *[24, 25]. A study conducted in Eritrea in 2001–2002 identified seven Anopheles species, three of which are potential malaria vectors [22, 26]. The same study also revealed one *Anopheles gambiae* infected with *Plasmodium falciparum* at an altitude above 2000 m [26]. The results of the present study indicate a broader expansion of Anopheles mosquitoes in the Maekel region, supporting the adaptive nature of these mosquitoes to diverse climatic and environmental conditions. Other studies in East Africa have illustrated the expansion of *Anopheles cinereus*, highlighting the ecological fitness for the Eritrean highlands. Studies in Mount Kenya have identified mosquitoes in high-altitude areas previously free from malaria [5]. The observed 2 °C increase in average temperature in the Kenyan highlands has been attributed to climate change. Similarly, the average temperature in the Eritrean highlands has increased by 1.72 °C [27], suggesting that climate change has been a primary driver of Anopheles mosquito expansion in the Maekel region since the 2001–2002 observations.

While the other Anopheles species identified in this study are less potent malaria vectors than *Anopheles gambiae*, their extensive habitat suitability is concerning. Several Anopheles species have been shown to transition to primary malaria vectors in various countries [28–30]. A study in northwest Ethiopia implicated *Anopheles cinereus* as a major malaria vector [28], and *Anopheles squamosus* carrying *Plasmodium falciparum* sporozoites was identified in Zambia [29]. The authors of the Zambian study concluded that the feeding behavior of *Anopheles squamosus* may help it evade current control measures, hindering malaria elimination [30]. Thus, identifying the specific Anopheles species responsible for indigenous malaria cases is crucial for malaria elimination efforts in the Maekel Region.

The findings of this study indicate that large areas of the Maekel region are highly suitable for various Anopheles mosquitoes, demonstrating their potential to overcome altitudinal barriers. Climate change is projected to further drive the expansion of Anopheles mosquitoes across the Maekel Region under both SSP3-3.7 and SSP5-8.5.

Although this study focuses on climatic suitability, a growing body of evidence indicates that anthropogenic land-use and land-cover change can substantially modify the spatial distribution and local abundance of *Anopheles* mosquitoes [31]. Urban built area in the Greater Asmara Area(GAA) has tripled over two decades [32], while irrigation activity increased by 65% within one subzone (Gala-Nefhi) between 2015 and 2018 [33]. Furthermore, the continued loss of natural forest and woodland cover may lead to localized warming and increased light penetration in larval habitats, potentially accelerating vector development rates. The expansion of irrigation and urban sprawl may have faciliated the observed distribution of species like *Anopheles gambiae* and *Anopheles cinereus*, suggesting that the vector shift is driven by a synergy between climatic change and the human-driven factors.

## Conclusion

This study indicates that many areas in the Maekel Region will be suitable habitats for Anopheles mosquitoes in the future. This finding poses a potential threat to the success of malaria control programs in the region. Therefore, enhanced surveillance and research are necessary to mitigate these risks. Additionally, a nationwide study is needed to map the distribution of Anopheles and Plasmodium species, enabling the development of a malaria control program to refine its strategies.

## Strengths and limitations

This study is the first of its kind in Eritrea to illustrate the potential risks of climate change and Anopheles adaptation and expansion via a robust analytical method. However, sample sizes for certain species are not optimal. While Maxent is robust with small sample sizes, additional samples enhance model accuracy. Moreover, this study haven’t included important variables such as LULC and soil modification as reliable, standardized future projections of those variables were difficult to source.

## Data Availability

No datasets were generated or analysed during the current study.
